# Test parameter selection affects the perceived antibacterial efficacy of a contact-killing coating developed to prevent prosthetic joint infection

**DOI:** 10.3389/fbioe.2026.1854828

**Published:** 2026-09-10

**Authors:** Julia L. van Agtmaal, Cynthia Calligaro, Nihal Engin Vrana, Sander M. J. van Kuijk, Tim J. M. Welting, Jacobus J. C. Arts

**Affiliations:** 1 Department of Orthopaedic Surgery, Research Institute CAPHRI, Maastricht University Medical Center, Maastricht, Netherlands; 2 SPARTHA Medical, Strasbourg, France; 3 Department of Clinical Epidemiology and Medical Technology Assessment, Maastricht University Medical Centre, Maastricht, Netherlands; 4 Orthopaedic Biomechanics, Department of Biomedical Engineering, Eindhoven University of Technology, Eindhoven, Netherlands

**Keywords:** antibacterial efficacy, coating, contact-killing, *in vitro*, prosthetic joint infection

## Abstract

**Introduction:**

Prosthetic joint infections occur in 1% -4% of total hip and knee arthroplasties. Many new treatment approaches to prevent bacterial adhesion and biofilm formation are under development. This study highlights the importance of methodological parameter selection and data processing in in vitro antibacterial testing by evaluating a contact-killing coating composed of poly-ε-lysine and hyaluronic acid (PEL-10/HA-1).

**Methods:**

Two broth compositions, inoculum volumes, and agitation speeds were evaluated to test bacterial adhesion on PEL-10/HA-1-coated and uncoated titanium alloy Ti6Al4V (Ti) samples. The samples were inoculated with 1 × 107 colony-forming units (CFU)/mL of Staphylococcus aureus (ATCC 25923). The inoculum was prepared in tryptic soy broth (TSB) or Mueller-Hinton broth (MHB) in volumes of 0.5 or 1 mL. The inoculated samples were incubated for 24 h at 60 or 120 RPM (0.05 or 0.2 g, respectively). After incubation, the attached CFU per sample were counted.

**Results:**

In MHB, a >5 log reduction in CFU/sample was observed for the coated samples compared to the uncoated ones. In TSB, reductions ranged from 0.4 to 3.5 log. A volume of 0.5 mL and an agitation speed of 120 RPM resulted in higher reductions in CFU/sample compared to 1 mL and 60 RPM, respectively. The main source of variation was the type of broth, followed by agitation speed and inoculum volume.

**Discussion:**

In conclusion, methodological choices in seemingly standard antibacterial efficacy testing protocols significantly influence the outcomes. These results underscore the critical need to carefully select and thoroughly document parameters to ensure the effectivity and reliability of testing antibacterial approaches.

## 1 Introduction

Although total hip and knee arthroplasties (THA and TKA) are generally highly successful procedures to restore function and relieve pain in people suffering from osteoarthritis, prosthetic joint infection (PJI) occurs in 1%–4% of these surgeries (van Agtmaal et al., 2025). Infections occur via direct inoculation, hematogenous seeding, or contiguous spread (Frigui et al., 2024; Diaz-Dilernia et al., 2026). Within a year after surgery, PJIs mainly result from contact or contamination of implants or tissues during surgery (Diaz-Dilernia et al., 2026). Superficial surgical site infections can worsen to reach the prosthesis and deeper tissues (Diaz-Dilernia et al., 2026). Late PJIs may happen if old wounds are disrupted or if hematogenous spread occurs during follow-up (Diaz-Dilernia et al., 2026; Dreikausen et al., 2023; Guarch-Pérez et al., 2023; Rak et al., 2019). PJI can lead to longer treatment and hospital stays, additional surgeries, functional impairments, reduced quality of life, and higher morbidity (Interventies, 2024; Parvizi et al., 2016; Davidson et al., 2019; Lakhani et al., 2024). The prevention and treatment of PJI mainly depend on antibiotics (Cao et al., 2024). Bacteria that attach to the implant itself or infect the surrounding tissue can develop into biofilms. Biofilms are an accumulation of bacteria surrounded by an extracellular matrix. Once mature, microaggregates of the biofilm or single bacteria can disperse to other areas (Arciola et al., 2018). The biofilm matrix, composed of polysaccharides, proteins, extracellular DNA, and lipids, encases the bacteria and limits antimicrobial penetration and effectiveness (Bhattacharya et al., 2015). Biofilms also protect bacteria from the immune system response, leading to persistent infections (Arciola et al., 2018). Bacteria within a biofilm can also enter a tolerant state, marked by reduced metabolic activity that in turn diminishes the activity of antibiotic targets (Brauner et al., 2016). As microorganisms become more resistant to antibiotics, and biofilm formation increases therapeutic insufficiency, alternative treatment and prevention options are needed (Commission, 2017). Many new treatment technologies are currently being developed to prevent or treat bacterial attachment and biofilm formation, such as (but not limited to) antimicrobial peptides, bacteriophage therapy, bioceramics, bioactive glass, induction heating, metal-based materials, polymers, and surface topography modifications (van Agtmaal et al., 2025). These new treatments aim to limit the need for antibiotics and thereby support antimicrobial stewardship.

Although such antibacterial approaches possess very different mechanisms of action, only three variants of the required standard testing method, namely, ISO 22196, ASTM E2180-18, and JIS Z 2801, are used for regulatory compliance and antibacterial efficacy testing (ISO, 2011; ASTM, 2018; Jis, 2000). These tests are simple, affordable, widely available, and ideal for screening technologies as a proof-of-concept test (Bento de Carvalho et al., 2024). Since contact between the bacterial suspension and the test material is guaranteed, contact-killing and release coatings can be evaluated using these standards (Sjollema et al., 2018). However, these tests are not suitable for anti-adhesion coatings, as bacteria from the suspension and bacteria attached to the material surface are recovered and quantified together as a single colony-forming units (CFU) count, making it impossible to distinguish reduced adhesion from bacterial killing. These standards are originally designed for plastics rather than medical implant applications, they do not specify a minimum log reduction in bacteria and leave room for specific adjustments. Such adjustments include incubation temperature and duration; initial bacterial concentration and volume; broth composition, concentration, and volume; humidity; airflow; surface topography; and bacterial strains and growth phases (Bento de Carvalho et al., 2024; Wiegand et al., 2018; Cunliffe et al., 2021). Additionally, different efficacies were observed for materials with intermediate antibacterial effects when tested in different laboratories (Bhattacharya et al., 2015). The parameters of ISO 22196, ASTM E2180-18, and JIS Z 2801 are optimized to maximize antibacterial performance, but do not accurately reflect clinical environments, potentially leading to exaggerated or erroneous efficacy claims (Bento de Carvalho et al., 2024; Cunliffe et al., 2021). Since emerging treatment technologies are based on various mechanisms of action, one antibacterial efficacy test is not expected to fit all. Additional testing methods are necessary to validate and fully understand the delivered antibacterial activity results (Bento de Carvalho et al., 2024).

Specific tests should be used to assess the effectiveness of antibacterial compound-releasing, contact-killing, or anti-adhesive properties of antibacterial approaches. Such tests can include agar zone of inhibition methods, complete submersion of the sample in the inoculum, methods involving a high surface area to volume ratio, adhesion techniques, and biofilm assays (Sjollema et al., 2018). Experimental protocols should be tailored to whether a technology is designed for prophylaxis or a therapeutic effect. Long-lasting PJI involves a higher microbial burden and tolerant biofilm populations that are less sensitive to treatment (Sjollema et al., 2018; Moriarty et al., 2014). Preventing direct postoperative PJI involves mainly intercepting planktonic bacteria, which require lower antimicrobial concentrations to kill than mature biofilms (Sjollema et al., 2018; Moriarty et al., 2014). Tests are further influenced by the components within the bacterial culture broth and supplements, such as tryptone or glucose, which may interact with antibacterial compounds. For example, by protein adsorption to the sample surface before or during evaluation (Sjollema et al., 2018; Moriarty et al., 2014). Since tests and antibacterial mechanisms vary, there is a gap in translating findings from *in vitro* to complex *in vivo* systems (Moriarty et al., 2014; Malone et al., 2017).

The influence of the choice of a specific antibacterial efficacy test was highlighted when testing the antibacterial activity of a novel polyelectrolyte contact-killing coating composed of poly-ε-lysine and hyaluronic acid (PEL-10/HA-1) for orthopedic implants *in vitro*. PEL-10 is contact-killing due to its positive charges, which disrupt the bacterial cell membrane (Hyldgaard et al., 2014; Lebaudy et al., 2023; Rodrigues et al., 2020; Tan et al., 2019), and HA-1 possesses antifouling properties (Gribova et al., 2020). Up to a 5-log reduction was observed in coated samples compared to uncoated ones, using the ISO 22196, ASTM E2180-18, and JIS Z 2801 standards for both *Staphylococcus aureus* and *Escherichia coli* (van Agtmaal et al., 2026). However, in a more challenging bacterial adhesion in suspension test, initial results varied significantly across the selected experimental variables, including broth, shaking settings, and inoculum volume (van Agtmaal et al., 2026). This study, therefore, aims to highlight the importance of parameter selection and data processing in antibacterial testing by evaluating the PEL-10/HA-1 coating, considering various media, inoculum sizes, and agitation speeds. The findings are intended to inform and guide parameter selection in future antibacterial testing.

## 2 Methods

### 2.1 Substrates

Titanium (Ti) samples were 5 mm thick with a diameter of 13.9 mm. The Ti alloy used was Ti6Al4V ELI (DePuy Synthes, Warsaw, Indiana, United States), conforming to ASTM F136 standards. All samples were cut from bar stock using wire EDM, grit blasted with alumina 25 grit to achieve a consistent surface roughness of approximately 3 µm Ra, and cleaned with deionized (DI) water. All samples were sterilized by autoclavation at 121 °C for 15 min.

### 2.2 Coating process

All samples were sonicated for 5 min in 70% ethanol and dried at room temperature. PEL solution was prepared at 10 mg/mL (PEL-10), and HA solution was prepared at 1 mg/mL (HA-1) in Tris-NaCl buffer (20–20 mM) at pH 7.4. These solutions were sprayed onto one side of the samples (250 μL/cm^2^), forming a thin coating through electrostatic interaction between the positively charged PEL-10 and the negatively charged HA-1. The samples were dried at room temperature before coating the other side. They were sterilized with UV light on both sides for 30 min. Coated and uncoated samples used in this study have been characterized previously (van Agtmaal et al., 2026).

### 2.3 Antibacterial activity tests

**FIGURE 1:**
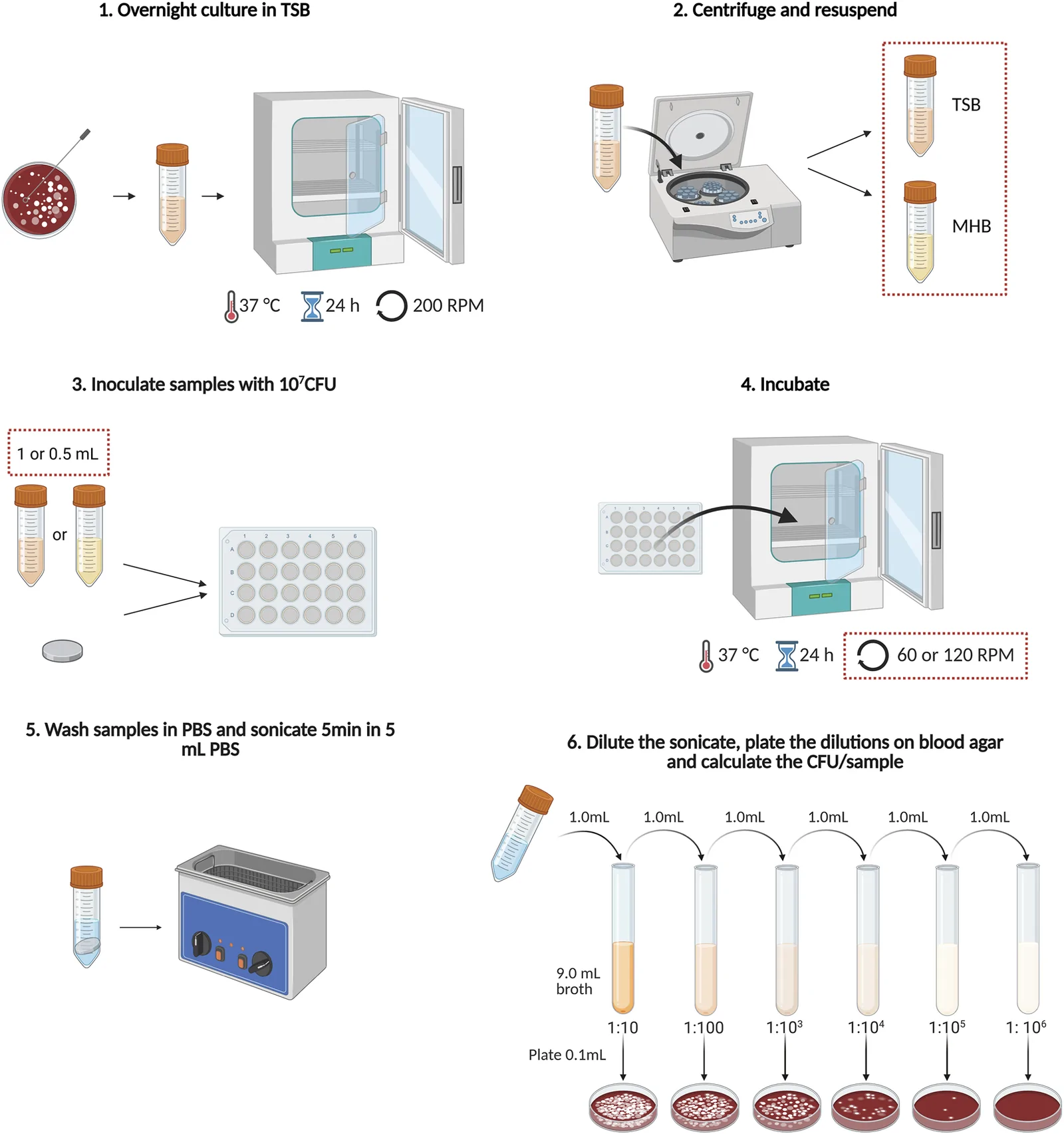
Overview of the setup of the bacterial adhesion test in suspension. In red dotted squares are the parameters visualized that have been changed per experiment. The bacterial suspension was suspended in TSB or MHB (step 2), 1 or 0.5 mL of this suspension was used (step 3), with both 10^7^ CFU, and incubation was at 60 or 120 RPM (step 4).

A bacterial suspension test with several variations in parameters was conducted to evaluate the effect of several test parameters on the PEL-10/HA-1 coating’s ability to prevent bacterial adhesion to the samples (Figure 1). Experiments were conducted following van Agtmaal et al. (van Agtmaal et al., 2026), with minor modifications. All procedures were performed under aseptic conditions using a laminar flow cabinet. Additionally, all reagents were autoclaved (high-pressure steam at 121 °C for 15 min). To achieve higher statistical power, all experiments were repeated three times.

*S. aureus* (ATCC 25923) was stored in 20% glycerol at −80 °C. An overnight axenic culture of *S. aureus* was prepared on a blood agar plate (Thermo Scientific, R01217) at 37 °C. From this axenic culture, 2-3 colonies were cultured (18–20 h, 37 °C, 200 rpm; New Brunswick Innova® 42 - M1335-0012, Eppendorf, Hamburg, Germany) in 5 mL tryptic soy broth (TSB; Sigma-Aldrich, 22092, composition in Table 1).

**TABLE 1:** Composition of the broths. Mueller Hinton Broth (BD, 211443) (Systems, 2009), and Tryptic Soy Broth (Sigma-Aldrich, 22092) (Aldrich, 2018).

Mueller hinton broth		Tryptic soy broth	
Ingredient	g/L	Ingredient	g/L
Beef extract Acid hydrolysate of casein Starch	3.0 17.5 1.5	Casein peptone (pancreatic) Soya peptone (papain digest.) Sodium chloride Dipotassium hydrogen phosphate Glucose	17.0 3.0 5.0 2.5 2.5

Half of the bacterial suspension was kept in TSB, the other half was centrifuged (3000RPM, 10 min) and resuspended in Mueller-Hinton broth (MHB; BD, 211443, composition in Table 1) (Figure 1, step 2). The suspension was diluted to the target inoculum concentration of ∼1 × 10^7^ CFU/mL using optical density (OD) measurements at 600 nm. The target inoculum was serially diluted, and 100 µL aliquots were plated in duplicate on blood agar plates (Thermo Scientific, R01217) to quantify the exact colony-forming units (CFU)/mL. Prior to the experiments, osmolarity (Freezepoint^TM^ Freezing Point Osmometer, ELITechGroup, Puteaux, France) and pH (pH Sensor InLab® Viscous, Mettler Toledo, Greifensee, Switzerland) of TSB and MHB were measured.

Three samples per group were placed in a 24-well plate. Samples were inoculated with 0.5 mL of the prepared inoculum or 1 mL of twice-diluted inoculum and incubated for 24 h at 37 °C (Figure 1, step 3). Half of the samples were incubated at 60 RPM and half at 120 RPM (Figure 1, step 4), representing a low and a higher level of agitation. Relative centrifugal force (RCF) is the force of acceleration applied to a sample due to shaking in the incubator, with RCF [g] = 1.12 × r × (RPM/1,000)^2^, where r represents the orbital radius (Crisci et al., 2024). Therefore, with an orbital diameter of 2.5 cm, 60 and 120 RPM are equal to 0.05 g and 0.2 g, respectively. After incubation, samples were washed with 2 mL PBS to remove non-adherent bacteria, and sonicated in 5 mL PBS for 5 min, serially diluted, and plated on blood agar plates in duplicate to count CFU after 24 h incubation at 37 °C.

### 2.4 Statistics

Statistical analysis was performed using GraphPad Prism 10.1.2 (GraphPad Software, Inc.) for Windows. Data was assumed to be normally distributed. To enhance the visualization of the differences between the coated and uncoated samples, the logarithm of all values was computed. These logarithmic values were then averaged, generating the geometric mean. The geometric mean of the coated samples was subtracted from the geometric mean of the uncoated samples, and the standard error of the mean (SEM) was calculated. On this data, a three-way ANOVA was performed, including multiple comparisons for means of cells that differ by only one factor, with factors broth (TSB or MHB), volume (0.5 or 1 mL), and RPM (60 or 120). Bonferroni’s correction for multiple comparisons was performed. A significance level of P < 0.05 was set.

## 3 Results

**FIGURE 2:**
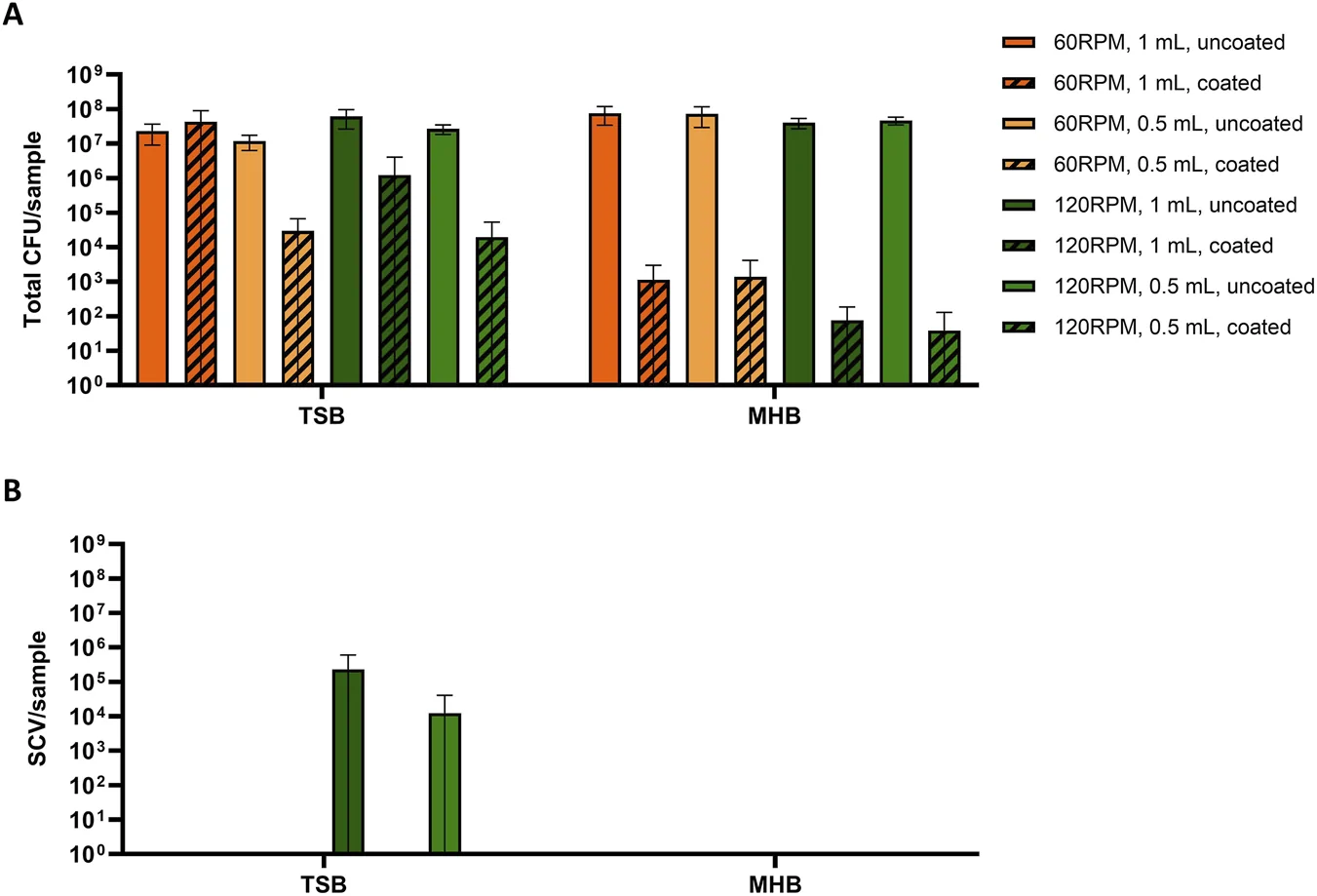
Results from the bacterial adhesion test using TSB or MHB, 1 mL or 0.5 mL, and 60 RPM or 120 RPM. Viable bacteria that adhered to the samples after 24 h of incubation were collected from the samples. The average starting inoculum was 1.80 ± 0.75 × 10^7^ CFU/mL. The striped bars represent coated samples, and the bars without stripes represent uncoated samples. Each bar represents the arithmetic mean ± SD (calculated by taking the log of the mean CFU/sample of three experiments, each including three samples (Supplementary Table S1). **(A)** Represents the normal-sized colonies plus the small colony variants (SCV), **(B)** represents only the SCV, the apparent sparsity reflects the restricted occurrence of SCVs among the tested conditions.

To test the effect of parameter selection on the antibacterial activity of the PEL-10/HA-1 coating, the coating was tested in a bacterial adhesion test in suspension. Variable parameters were MHB or TSB, 0.5 mL or 1 mL, and shaking at 60 RPM or 120 RPM (Figure 2A). The average starting inoculum was 1.80 ± 0.75 × 10^7^ CFU/mL. After 24 h of sample incubation in the bacterial inoculum, the CFU for the uncoated samples ranged from 1.19 × 10^7^ to 7.63 × 10^7^ CFU/sample. These values are all within 0.64 log units and are of the same magnitude, indicating that the test parameters had little effect on the bacterial attachment to the uncoated samples. For the coated samples, it ranged from 3.89 × 10^1^ to 4.26 × 10^7^ CFU/sample, indicating a significant difference between the experimental parameters. Remarkably, at 120 RPM in TSB for both 0.5 and 1 mL, small colony variants (SCV) were visible on the blood agar plates of the coated samples (Figure 2B; Supplementary Figure S1). For the 0.5 mL samples, the SCV made up 37% of the total CFU, and for 1 mL, 35%. However, this only changed the total CFU 0.43 log for 0.5 mL and 0.09 log for 1 mL. At 60 RPM and in MHB, no SCV were found.

**FIGURE 3:**
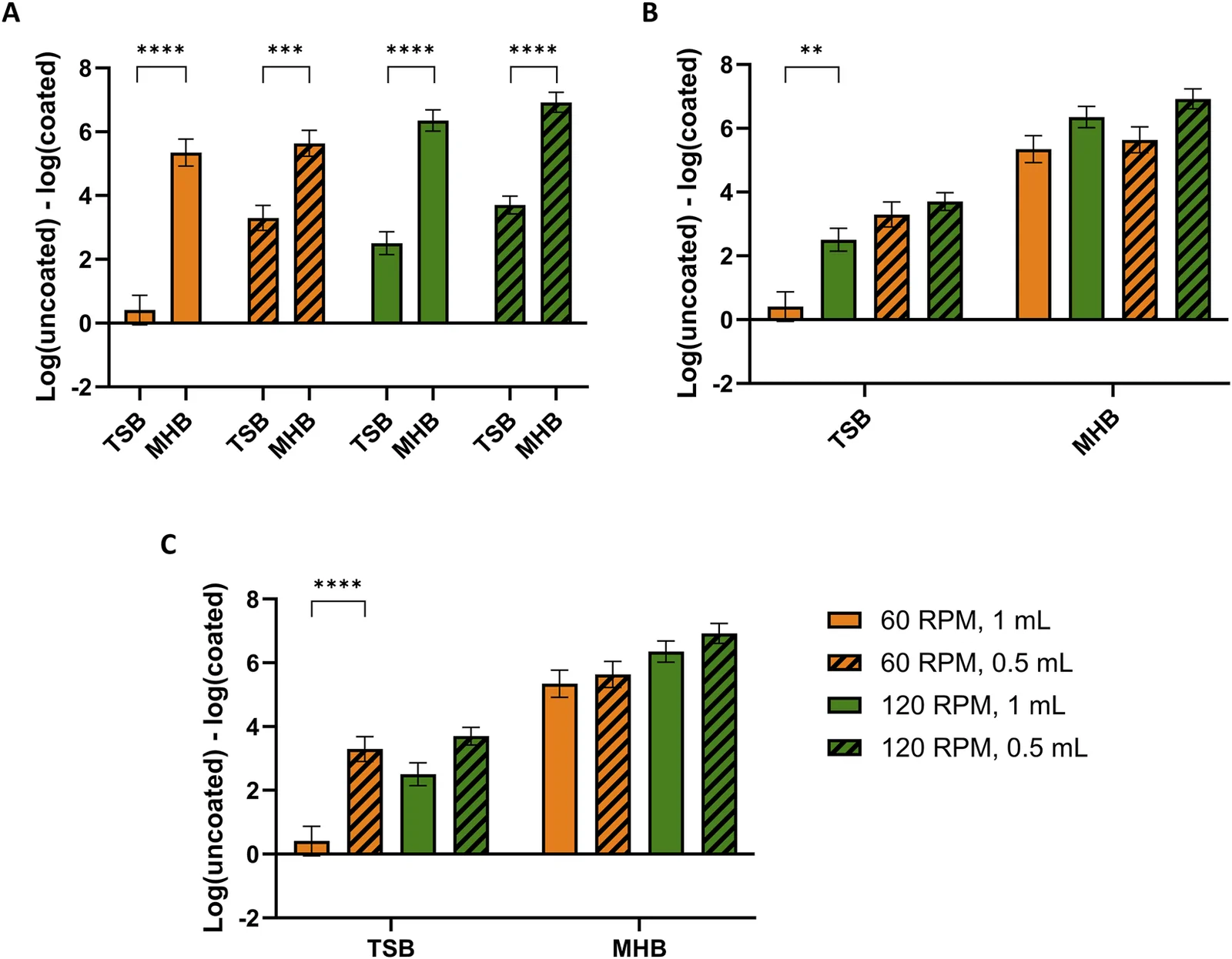
Differences between the mean logarithmic values of the uncoated minus the mean logarithmic values of the coated samples. Each bar represents the geometric mean ± the SEM. Statistical differences were found with three-way ANOVA, including multiple comparisons, with factors broth (TSB or MHB), volume (0.5 or 1 mL), and RPM (60 or 120), *p < 0.05, **p < 0.01, and ****P < 0.0001. Multiple comparisons included comparisons of means of cells that differ by only one factor, and are visualized based on broth **(A)**, RPM **(B)**, and inoculum volume **(C)**.

To enhance visualization of the antibacterial effect, the difference between the mean log CFU/sample of the uncoated and coated samples was calculated, representing the geometric mean (Figure 3; Supplementary Table S2). The differences between the coated and uncoated samples represented by the arithmetic means in Figure 2 seem smaller compared to how they are represented as geometric means in Figure 3. Geometric mean results showed that using TSB resulted in significantly lower log differences between uncoated and coated samples across both volumes and RPM settings compared to MHB (Figure 3A). Additionally, lower agitation speeds (60 RPM vs. 120 RPM) generally produced lower log differences, however, only statistically significant only for TSB with 1 mL test volume (Figure 3B). For volume, 1 mL consistently yielded lower log differences than 0.5 mL, however, only statistically different for TSB at 60 RPM (Figure 3C). A three-way ANOVA assessed the effects of broth type, RPM, and volume on these results. It revealed that the primary source of variation was primarily due to the broth (>60% of the variation) (Table 2). However, while contributing to a lesser extent, RPM, inoculum volume, and broth x volume were also statistically significant sources of variation.

**TABLE 2:** Source of variation calculated with three-way ANOVA. The main effects of the independent factors, two-way interactions, and three-way interaction is examined.

Source of variation	% of total variation	P value
Broth	60.23	<0.0001
RPM	6.74	<0.0001
Volume	7.14	<0.0001
Broth x RPM	0.01	0.8503
Broth x Volume	3.04	0.0035
RPM x Volume	0.58	0.1889
Broth x RPM x Volume	1.12	0.0696

The pH and osmolality affect bacterial growth, bacterial metabolic activity, and cellular stress responses (Müller et al., 2024; Valero et al., 2009). Therefore, the pH and osmolality were measured for both the MHB and TSB (Table 3) across all experiment rounds to determine if the values stayed within the preferred range for bacterial growth. For pH, the range is 4.0–10.0, with an optimum of 6.0–7.0 (Valero et al., 2009). Blood serum osmolality is 285–295 mOsm/kg (Im et al., 2017). For laboratory strains, the ideal is generally the lowest osmotic pressure that does not cause cell lysis, with a reduction in bacterial growth observed at >1,000 mOsm/kg (Cebrián et al., 2015). The pH showed no statistically significant difference between the two broths. However, the osmolality of TSB (300.0 ± 5.3 mOsm/kg) was significantly lower than that of MHB (314.3 ± 0.6 mOsm/kg).

**TABLE 3:** For each repeat of the bacterial adhesion test, a separate solution of MHB and TSB was made. The pH and Osmolality were measured in these solutions. A t-test was performed to assess the differences between the two broths.

Parameter	MHB	TSB	p-value
pH (calibrated to 25 °C)	7.40 ± 0.07	7.29 ± 0.14	0.2858
Osmolality [mOsm/kg]	314.3 ± 0.6	300.0 ± 5.3	0.0096

## 4 Discussion

The current study demonstrated that altering methodological test parameters (focusing on broth type, inoculum volume, and shaking settings) in standard protocols for evaluating antibacterial approaches can influence experimental outcomes. These protocols are perceived as robust benchmarks, but changes to these parameters can affect the interpretation of results. Minor deviations in methodological choices can significantly affect the outcomes of antibacterial effectiveness studies. These insights challenge the perceived robustness and reproducibility of current test standards. This study demonstrated that test parameter selection significantly influences measured antibacterial efficacy, highlighting the need for careful parameter selection and transparent documentation to avoid conclusions based on methodological artifacts rather than true coating performance.

The bacterial attachment to uncoated and PEL-10/HA-1-coated Ti samples was tested using an inoculum of 10^7^ CFU and 24 h incubation. The differences between MHB and TSB, 0.5 and 1 mL bacterial suspension, and incubation at 60 and 120 RPM were systematically investigated. In most studies, antibacterial activity is considered clinically relevant when there is a 2–3 log, or 99%–99.9% reduction in CFU between the coated and uncoated samples (Jis, 2000; Sjollema et al., 2018; Moriarty et al., 2014; Cunliffe et al., 2021). This reduction was observed across all tests in MHB, but varied from 0.41 to 3.29 log in TSB, indicating that selecting specific parameters can vary an antibacterial technology from seemingly clinically relevant to not. The graph with absolute values shows the arithmetic mean CFU/sample, making the log difference between coated and uncoated samples appear smaller than the geometric mean. The calculated mean is often not disclosed in studies. However, it does imply different clinical relevance of the same results. Calculating the log of each value and then averaging this (geometric mean) is the most statistically appropriate way for bacterial data, as it tends to be log-normally distributed and positively skewed. Therefore, the arithmetic mean can mislead, as the mean will be pulled towards higher values, leading to different interpretations (Gao and Martos, 2019).

Broth composition is known to influence the proliferation and metabolic activity of specific bacterial strains, and the effectiveness of antibacterial techniques (Sjollema et al., 2018; Paleczny et al., 2021; Lopes et al., 2023; Sharifi et al., 2022). The substantial impact of the broth is reflected by 60% of the total variation in this study being attributed to the broth. Both osmolality and pH values of the two broths were within the optimal range for *S. aureus* proliferation (Müller et al., 2024; Valero et al., 2009). Unlike MHB, TSB is buffered with dipotassium hydrogen phosphate to maintain a stable pH and is stabilized with sodium chloride for osmotic balance, thereby ensuring stability throughout the experiment. In this study, bacterial attachment to uncoated samples was similar for both broths. Contrary, Paleczny et al. (2021) compared TSB (with or without 1% glucose) to Dulbecco’s Modified Eagle’s Medium (DMEM). They found that while two ATCC and ten clinical *S. aureus* strains formed similar biofilm levels across all media, both metabolic activity and biomass levels were lower in DMEM. Lopes et al. (2023) analyzed bacterial proliferation in Brain Heart Infusion (BHI), MHB, and TSB, supplemented with 0%–5% glucose. The highest bacterial proliferation of *S. aureus* ATCC 25923 was found in TSB containing 2% glucose and in BHI with 5% glucose (Lopes et al., 2023). In contrast, the optimal proliferation in MHB occurred without any added glucose (Lopes et al., 2023). For *E. coli* ATCC 25922, the optimal growth medium, based on both biomass and CFU counts, was BHI broth supplemented with 5% glucose (Lopes et al., 2023). MHB without glucose is best for *Pseudomonas aeruginosa* (ATCC 47085), as this species does not prioritize glucose consumption (Lopes et al., 2023). Sharifi et al. (2022) found *S. aureus* (ATCC 25923 and two clinical isolates) biofilm growth to be best in TSB, compared to Nutrient Broth (NB), MHB, and BHI, and found that supplementation with 1% glucose was optimal.

A clear statistical difference in the antibacterial effect of the PEL-10/HA-1 coating was found between using MHB and TSB. This may have been caused by the richer nutritional composition of TSB compared to MHB (Hulankova, 2022). TSB has glucose readily available as a carbon source, while in MHB, only a small amount of dextrose is available as a carbon source due to potential starch hydrolysis during autoclaving (Aldrich, 2018; Systems, 2009). MHB includes an acid hydrolysate of casein, and strong acid hydrolysis is known to degrade certain sensitive amino acids, resulting in a changed amino acid profile (Systems, 2009; Internacional, 2026; Wang et al., 2013). The casein peptone in TSB is produced using enzymatic digestion, preserving amino acids in peptide form (Aldrich, 2018; Internacional, 2026; Wang et al., 2013). The different presentation of amino acids and the available glucose in TSB might stimulate microbial growth more than MHB (Dai et al., 2021; Idrees et al., 2020). The availability of carbon sources from glucose and amino acids in peptide form in TSB might explain why bacteria recover better from antibacterial stresses in TSB compared to MHB. Moreover, PEL and HA can bind to the starch in MHB. This interaction has been shown to improve antibacterial effects, compared to testing in TSB and BHI broth (Hulankova, 2022; Yoon et al., 2023; Zhang et al., 2015).

Paleczny et al. (2021) also demonstrated that the antibacterial activity of several compounds varies depending on the broth that is used. Some compounds had a lower minimal inhibition concentration (MIC) in DMEM due to reduced bacterial metabolism and proliferation (Paleczny et al., 2021). Others had a higher MIC because amino acids bound to the compounds, reducing their availability (Paleczny et al., 2021). Lastly, for some compounds, the antibacterial effect depended on bacterial strain sensitivity (Paleczny et al., 2021). Glucose was not found to affect the antibacterials (Paleczny et al., 2021). The antibacterial effect of tigecycline was tested in five different broths, with and without Tween 80, and all combinations demonstrated different antibacterial activities (Usacheva et al., 2014). Together, these results demonstrate a significant effect of the choice of broth and supplements on the measured antibacterial activity of a compound. Researchers should therefore consider testing antibacterial compounds in several broths to ensure antibacterial efficacy across multiple setups. Specific metabolic traits from a bacterial strain and species might further direct the choice of a specific broth or supplements.

The PEL-10/HA-1 coating demonstrated a higher antibacterial effect at 120 RPM than at 60 RPM. Although flow rate and shear forces in flow displacement systems have been studied, most adhesion studies only report the RPM, which cannot be compared between different incubators. RCF should be documented for comparison. Higher agitation facilitates bacterial adhesion by elevated encounter rates with the sample surface (Saur et al., 2017; Juergensmeyer et al., 2007; Lin et al., 2021; Alves et al., 2020). However, high rotation rates also increase the hydrodynamic drag on attached cells, causing cells to slide and roll, thereby preventing adhesion or breaking initial, reversible attachments (Saur et al., 2017; Alves et al., 2020; Staats et al., 2022). Also, bacteria with morphological changes due to antimicrobials can have impaired initial attachment and transition to irreversible binding at higher RPM compared to static assays (Fonseca and Sousa, 2007). These observations imply that increased RPMs are associated with more frequent cell-coating contact, and that higher drag forces could hinder stable initial attachment, potentially affecting adhesion efficiency (Zühlke et al., 2016). Higher RPM also increases oxygenation. Although *S. aureus* is facultatively anaerobic, proliferation rates are four times higher in aerobic incubation due to metabolic changes (Zühlke et al., 2016). Also, oxygen can influence bacteriostatic properties and the MIC and MBC values of antimicrobials on *S. aureus*, both positively and negatively (Park et al., 1992).

The PEL-10/HA-1 coating performed better in 0.5 mL compared to 1 mL inoculum. A lower volume with the same number of CFU facilitates easier contact with the contact-killing coating (Sjollema et al., 2018). For flow displacement systems, the Smoluchowski-Levich approximation estimates the mass transport from the suspension to the sample surface. In these systems, a higher bacterial concentration results in more bacteria reaching the surface, increasing contact with the coating and thus bacterial death (Alves et al., 2020; Busscher and Van Der Mei, 2006). A long-term side effect might be a layer of dead cells and debris, which can serve as anchoring points for cell adhesion and provide protection from the coating (Alves et al., 2020). Additionally, higher bacterial concentrations have been shown to increase the peak number of adhering cells and shorten the reversible adhesion process, possibly leading to more interaction with the coating (Wang et al., 2020). This demonstrates not only the importance of the absolute CFU challenge number, but also the inoculum volume. The chosen inoculum concentration and volume should be considered based on the antibacterial working mechanism, clinical indication and implementation.

SCVs are a slow-growing bacterial subpopulation with varying sizes, typically forming colonies approximately 1/10th of the size of the original strain on agar plates (Bui and Kidd, 2015; Askar et al., 2018). Non-stable SCV demonstrate colony size heterogeneity on agar plates. Their size varies, influenced by developmental and metabolic pathways, and genetic mutations from stress, including nutrient limitations, or sublethal concentrations of antimicrobials (Bui and Kidd, 2015; Askar et al., 2018; Bayston et al., 2007). They are quasi-dormant, can revert to their parental cell type, and are more tolerant to antimicrobials (Bui and Kidd, 2015; Cao et al., 2017). Non-stable SCVs demonstrate colony size heterogeneity on SCVs were formed only at 120 RPM in TSB. Standard culture methods are optimized for rapidly growing, normal-colony bacteria, making SCV counting challenging. However, the antibacterial activity might differ when including SCVs. SCV are considered to have disrupted electron transport chains, altering the respiratory chain and cross-membrane potential, possibly altering electrostatic interactions with PEL (Cao et al., 2017). Mutations in the respiratory chain result in upregulated arginine deiminase and pyruvate fermentation pathways to produce ATP (Cao et al., 2017). TSB is richer in arginine than MHB due to the Soya peptone, and its glucose can be turned into pyruvate, possibly explaining SCV growth in TSB but not in MHB. SCV growth at 120 RPM might be caused by oxygenation or other metabolic interactions. While oxygen depletion and oxidative stress have both been demonstrated to result in SCV (Besse et al., 2023; Painter et al., 2015), the exact link between oxygenation and SCV formation is currently unknown. Because SCV exhibit slow growth, necessitating up to 72 h of incubation for detection, they are frequently overlooked in standard antibacterial tests designed for fast-growing aerobic bacteria (Askar et al., 2018; Precit et al., 2016). It is possible that SCVs in MHB or at 60 RPM promoted SCV formation, requiring extended incubation periods or alternative growth conditions for identification. Given their clinical relevance, the specific influence of test parameters on SCV remains an important topic for further investigation.

Standard tests like ISO or adhesion are useful initial assessments of antibacterial technology, but models with longer durations, dynamic systems or better physiological representation may better evaluate real clinical performance. Unlike static 24-h assays, a 5-day *in vitro* study demonstrated that fluctuating antibiotic concentrations led to significant differences in bacterial survival over time (Broussou et al., 2019). For a PJI treatment model, bacteria were grown for 7 days before exposure, reflecting the clinical delay (Verheul et al., 2024; Verheul et al., 2025). Dynamic models like the CDC bioreactor simulate *in vivo* conditions with nutrient flow, waste removal, shear forces, and adjustable fluid volume (Ramachandra et al., 2024; Morco et al., 2022). Flow perfusion models control mass transport and can be tailored for specific applications, with inoculation levels changing over time (Sjollema et al., 2018; Ashton et al., 2019). Synovial fluid or Roswell Park Memorial Institute (RPMI) medium models could better mimic the joint microenvironment (De Bleeckere et al., 2025; Bleick et al., 2025). Compared to standard media, bacteria in synthetic synovial fluid have shown reduced antibiotic susceptibility (De Bleeckere et al., 2025). Antibiotics and implants also interact with blood and marrow, where blood components impair antibiotic efficacy by binding or interference (Nussbaumer-Pröll et al., 2019; Ndieyira et al., 2014; Talebpour et al., 2025).

The question remains how *in vitro* results, which differ due to parameter selection, can be translated to the *in vivo* situation. *In vitro* test methods lack complexity, but also, the *in vivo* situation varies per animal, and bacterial strains can react differently *in vitro* and *in vivo* (Moriarty et al., 2014; Moriarty et al., 2019). In addition, *in vivo* results can differ between species due to pharmacokinetic profiles, anatomical, and physiological differences (Shi et al., 2019). *In vitro* parameters should be chosen in such a manner that they mimic the *in vivo* situation as closely as possible. However, many seemingly small choices can influence the *in vitro* results. In this respect, parameters such as humidity, contact time, airflow, surface topography, and bacterial growth phase at inoculation can influence outcomes (Cunliffe et al., 2021; Zhou and Li, 2015). Although these were not systematically varied in the present investigation, they should be considered in future test standardization efforts.

### 4.1 Conclusion

Comprehensive *in vitro* studies are essential for understanding antibacterial efficacy and guiding translational research. Key methodological choices significantly impact the assessment of antibacterial effectiveness. This study demonstrates the importance of selecting and documenting parameters when testing new antibacterial approaches. Without clear parameters, comparisons between studies become unreliable, and the true antibacterial efficacy will be left unknown. ISO and adhesion tests are suitable initial steps in evaluating antibacterial approaches. However, a comprehensive evaluation using multiple antibacterial tests is essential to assess and characterize antibacterial technology thoroughly. While these *in vitro* assays may not fully replicate *in vivo* or clinical conditions, they form an essential foundation for understanding antibacterial efficacy and guiding subsequent translational research. Where *in vivo* studies must follow ARRIVE guidelines (ARRIVE guidelines, 2024) in their documentation to enhance standardization for reproducibility and comparison, *in vitro* studies should also adhere to certain documentation requirements. Bacterial strains, inoculum preparation, broth type and supplements, incubation settings, and sample processing should all be clearly documented. Regulatory bodies and societies such as the European Bone and Joint Infection Society (EBJIS) and journals should enforce such documentation. Methodological choices significantly influence the outcomes of antibacterial effectiveness studies. The results of this study and available literature emphasize the importance of selecting and properly documenting parameters when testing the efficacy of new antibacterial approaches.

## Data Availability

The raw data supporting the conclusions of this article will be made available by the authors, without undue reservation.
