# Supplementary material for: Test parameter selection affects the perceived antibacterial efficacy of a contact-killing coating developed to prevent prosthetic joint infection
Source: Front Bioeng Biotechnol. 2026 Sep 10;14:1854828. doi: 10.3389/fbioe.2026.1854828 (PMC13601291; doi:10.3389/fbioe.2026.1854828)
Supplement: Supplementary file 1 [file Supplementaryfile1.docx]

Supplementary Material

# 1 Supplementary Data

**Supplementary Figure 1**. Example of small colony variants (SCV) formed after 24h incubation of titanium samples with the PEL-10/HA-1 coating in 1 mL of bacterial suspension at 120 RPM in TSB. Diameter agarplate is 90 mm.

**Supplementary Table 1.** The logarithmic values of the mean of the uncoated CFU/sample minus the logarithmic value of the mean of the coated CFU/sample within one group (arithmetic mean).

|  | TSB | | MHB | |
| --- | --- | --- | --- | --- |
|  | 1 mL | 0.5 mL | 1 mL | 0.5 mL |
| 60 RPM | -0.27 | 2.60 | 4.82 | 4.72 |
| 120 RPM | 1.69 | 3.13 | 5.73 | 6.08 |

**Supplementary Table 2.** The mean of the logarithmic values of the uncoated CFU/sample minus the mean of the logarithmic values of the coated CFU/sample within one group (geometric mean).

|  | TSB | | MHB | |
| --- | --- | --- | --- | --- |
|  | 1 mL | 0.5 mL | 1 mL | 0.5 mL |
| 60 RPM | 0.41 | 3.29 | 5.34 | 5.64 |
| 120 RPM | 2.50 | 3.70 | 6.36 | 6.92 |
